# Supplementary material for: Structure and dynamics of a multidomain nitric oxide synthase regulated by a C2 domain
Source: Sci Adv. 2026 Feb 20;12(8):eaeb4529. doi: 10.1126/sciadv.aeb4529 (PMC12922734; doi:10.1126/sciadv.aeb4529)
Supplement: Supplementary file 1 — Legend for movie S1 Tables S1 to S5 Figs. S1 to S19 [file sciadv.aeb4529_sm.pdf]

Science Advances
AAAS

Supplementary Materials for

Structure and dynamics of a multidomain nitric oxide synthase regulated by a C2 domain

Dhruva Nair and Brian R. Crane

Corresponding author: Brian R. Crane, bc69@cornell.edu

Sci. Adv. **12**, eaeb4529 (2026)
DOI: 10.1126/sciadv.aeb4529

The PDF file includes:

Legend for movie S1
Tables S1 to S5
Figs. S1 to S19

Other Supplementary Material for this manuscript includes the following:

Movie S1

Supplemental Movies

Movie S1. 3D Variability analysis of one subunit of the syNOS asymmetric dimer showing the breathing motion of NOS_{Red} as a unit relative to motion about the NOS_{Oxy} interface.

Supplementary Tables

Dataset	Conditions	Class 1	Class 2
1<br>2	GFB1	Symmetric Dimer (NOS-Sym)	Asymmetric Dimer (NOS-Asym)
3	GFB1 + 1mM Ca^{2+}/ 1mM L-arg / BH_4*	Asymmetric Dimer (NOS-Asym)	Turnover ($NOS\text{-}TO_L$)
4	GFB1 + 1mM Ca^{2+}/ 1mM L-arg / BH_4*	Asymmetric Dimer (NOS-Asym)	Turnover ($NOS\text{-}TO_T$)
5	GFB1 + 1mM Ca^{2+}/1mM L-arg/ BH_4* / 1mM NADPH	Monomer (NOS-Mon)	none

GFB1: 25 mM HEPES (pH 7.5), 150 mM NaCl, 1 mM TCEP, 10% glycerol and 10 µM BH_4.

***Additional 25 µM BH_4 was supplemented.

Table S1. Description of datasets, conditions and refined classes.

Inactive				
**Residue 1**	**Residue 2**	**Intra Cα- Cα**	**Inter Cα- Cα**	**XlinkX Score**
**FNR - FNR**				
1111	1017	**<u>8</u>**	143	124
1366	1447(CTT)	**<u>20</u>**	117	118
1053	1463(CTT)	**<u>40</u>**	121	75
1366	1053(CTT)	**<u>21</u>**	138	52
**Interdomain**				
191(pGlb)	538(Oxy)	**<u>17</u>**	**<u>24</u>**	78

Turnover				
**Residue 1**	**Residue 2**	**Intra Cα- Cα**	**Inter Cα- Cα**	**XlinkX Score**
**FNR-FNR**				
1019	1111	**<u>6</u>**	149	222
1111	1017	**<u>8</u>**	143	86
**Interdomain**				
196(pGlb)	538(Oxy)	**<u>16</u>**	**<u>31</u>**	97
191(pGlb)	538(Oxy)	**<u>17</u>**	**<u>24</u>**	78
470(Glb)	481(Oxy)	**<u>17</u>**	57	61
473(Glb)	481(Oxy)	**<u>12</u>**	58	50
196(pGlb)	481(Oxy)	**<u>24</u>**	44	48
413(Glb)	592(Oxy)	**<u>19</u>**	79	45
1017(FNR/L3)	994(Fld)	**<u>23</u>**	120	41
470(Glb)	538(Oxy)	40	55	41

Table S2. Identified and validated DSBU crosslinks. Reasonable distances based on the cryo-EM structures are bolded and underlined. PDB:9Q15 was used to estimate distances.

Dataset		Ca2+/L-Arginine			Ca^{2+}/L-Arginine/NADPH
**State**	**Symmetric Dimer**	**Asymmetric**	**Turnover**	**Asymmetric (Composite)**	**Monomer**
			**Data Collection and Processing**		
**Microscope**	Arctica		Krios		Krios
**Voltage (keV)**	200		300		300
**Detector**	K3		Gatan K3		Falcon4EC
**Nominal magnification**	63,000		105,000		105,000
**Data Acquisition Software**	Serial EM		Leginon		Leginon
**Electron dose (e–/Å2)**	50		50.5		44.95
**Pixel Size (Å) (binned)**	1.31		0.422(0.844)		0.7304
**Defocus range (µm)**	0.5-2		0.4-2.1		0.4-2.2
**Number of movies (#)**	1214 + 4055		5019		9036
**Number of particles**	131,000	70,944	61,553	70,944	45,964
**Symmetry imposed**	C2	C1	C1	C1	C1
**Resolution (Å)**	3.95	3.16	3.69	3.16	3.09
**FSC threshold**	0.143	0.143	0.143	0.143	0.143
			**Refinement**		
**Initial model used**	PDB 9Q15	PDB 9Q15	PDB 9Q15	Alphafold	PDB 9Q15
**Non-hydrogen atoms**	22712	16319	11268	16319	11404
**Protein residues**	2796	2001	1358	2001	1398
**Ligand(#)**	Heme(4)<br>FAD(2)<br>FMN(2)	Heme(2)<br>FAD(1)<br>FMN(1)	Heme(4)	Heme(4)<br>FAD(1)<br>FMN(1)	Heme(2) FAD(1)<br>FMN(1) NADPH(1)
**RMS Deviation**					
**Bond lengths (Å)**	0.02	0.005	0.003	0.003	0.003
**Bond angles (°)**	1.55	0.55	0.574	0.485	0.519
**MolProbity score**	1.54	1.58	1.5	1.74	1.54
**Clashscore**	2.18	3.49	4.65	4.66	4.93
**Poor rotamers (%)**	2.08	1.63	0.93	1.8	0.91
**Ramachandran Favored (%)**	95.1	96.0	96.1	95.5	96.0
**Ramachandran Allowed (%)**	4.6	3.9	3.7	4.3	3.8
**Ramachandran Disallowed (%)**	0.3	0.2	0.2	0.2	0.2
**Fit to map (CCmask)**	0.8	0.79	0.75	0.81	0.82
**EMDB (maps)**	EMD-72088	EMD-72110	EMD-72109	EMD-72104, EMD-72105,EMD-72116	EMD-72087
**PDB (model)**	9Q06	9Q0Y	9Q0X	9Q15	9Q05

Table S3. Cryo-EM data collection, refinement, and validation statistics.

Primer Name	Primer	Cloning Approach
pet28_twin_strep_syNOS_fwd	tcgagcaccaccaccacc	Gibson/NEB HiFi
pet28_twin_strep_syNOS_rev	catatgtcctccggattggaagtac	Gibson/NEB HiFi
syNOS_fwd	tccaatccggaggacatatgatgcttgtcaacgactctc	Gibson/NEB HiFi
syNOS_rev	gtggtggtggtggtgctcgagctacaagttggctagcc	Gibson/NEB HiFi
Hptg_fwd_PAYC_mcs1	TAATGCTTAAGTCGAACAG	Gibson/NEB HiFi
Hptg_rev_PAYC_mcs1	GGTATATCTCCTTATTAAAGTTAAAC	Gibson/NEB HiFi
Hptg_fwd	CTTTAATAAGGAGATATACCATGAAAGGACAAGAAACTCGTG	Gibson/NEB HiFi
Hptg_rev	TCTGTTCGACTTAAGCATTATCAGGAAACCAGCAGCTG	Gibson/NEB HiFi
gapa_fwd_pAYC_mcs2	TAATTAACCTAGGCTGCTG	Gibson/NEB HiFi
gapa_rev_pAYC_mcs2	ATGTATATCTCCTTCTTATACTTAAC	Gibson/NEB HiFi
gapa_fwd	TATAAGAAGGAGATATACATATGACTATCAAAGTAGGTATCAAC	Gibson/NEB HiFi
gapa_rev	GCAGCAGCCTAGGTTAATTATTATTTGGAGATGTGAGC	Gibson/NEB HiFi
pet28_tws_delc2_fwd	ctcgagcaccaccaccac	Gibson/NEB HiFi
pet28_tws_delc2rev	catatgtcctccggattggaag	Gibson/NEB HiFi
syNOSdelc2_178_1468_fwd	tccaatccggaggacatatgacctactcagaagccgtc	Gibson/NEB HiFi
syNOSdelc2_178_1468_fwd	tggtggtggtggtgctcgagctacaagttggctagccatttttc	Gibson/NEB HiFi
pet28_tws_delctt_fwd	tagctcgagcaccaccac	Gibson/NEB HiFi
pet28_tws_delctt_rev	catatgtcctccggattggaag	Gibson/NEB HiFi
syNOS_delctt1_1_1440_fwd	tccaatccggaggacatatgatgcttgtcaacgactctc	Gibson/NEB HiFi
syNOS_delctt1_1_1440_fwd	tggtggtggtgctcgagctagtagttgtctttctccacttg	Gibson/NEB HiFi
syNOS_K1412E_fwd	GCAAATTGCTgaaACAGAAGGAG	KLD
syNOS_K1412E_rev	ATAAATACCTCAAACACATTG	KLD
syNOS_T953A_fwd	GATCGGCAGCgcgGTCTATGAAC	KLD
syNOS_T953A_rev	CCTAGTACGGAATAGTTGAG	KLD
syNOS_K987A_fwd	CGACGAAATCgcgGGACAAGCCG	KLD
syNOS_K987A_rev	CCTTTATGTAGGGGAAC	KLD

Table S4. Primers used in the study with the cloning approach (left).

Domain	Couple	Potential (mV)	n	Calibrating dye (mV)
Globin (337-467)	Fe^{3+}/Fe^{2+}	-250 ± 20	1	safranin T (-289, 12.5μM)
Fld (856-1027)	ox/hq	-173 ± 3	2	cresyl violet (-177.0, 60 μM)
FNR (1027-1468)	ox/hq	-290 ± 13	2	safranin T (-289, 12.5 μM)

Table S5. Reduction potentials ($E^{0'}$) for $syNOS_{Glb}$, $syNOS_{Fld}$ and $syNOS_{FNR}$ vs. the Normal Hydrogen Electrode (NHE). For $syNOS_{Glb}$ the single electron reduction (n_e=1) from Fe^{3+} to Fe^{2+} was measured whereas for the flavin-containing domains a midpoint potential for the 2-electron reduction (n_e=2) from oxidized (ox) to hydroquinone (hq) was measured as no spectral signature for the semiquinone was apparent in the titrations. (n = 3 for all measurements)

Supplementary Figures

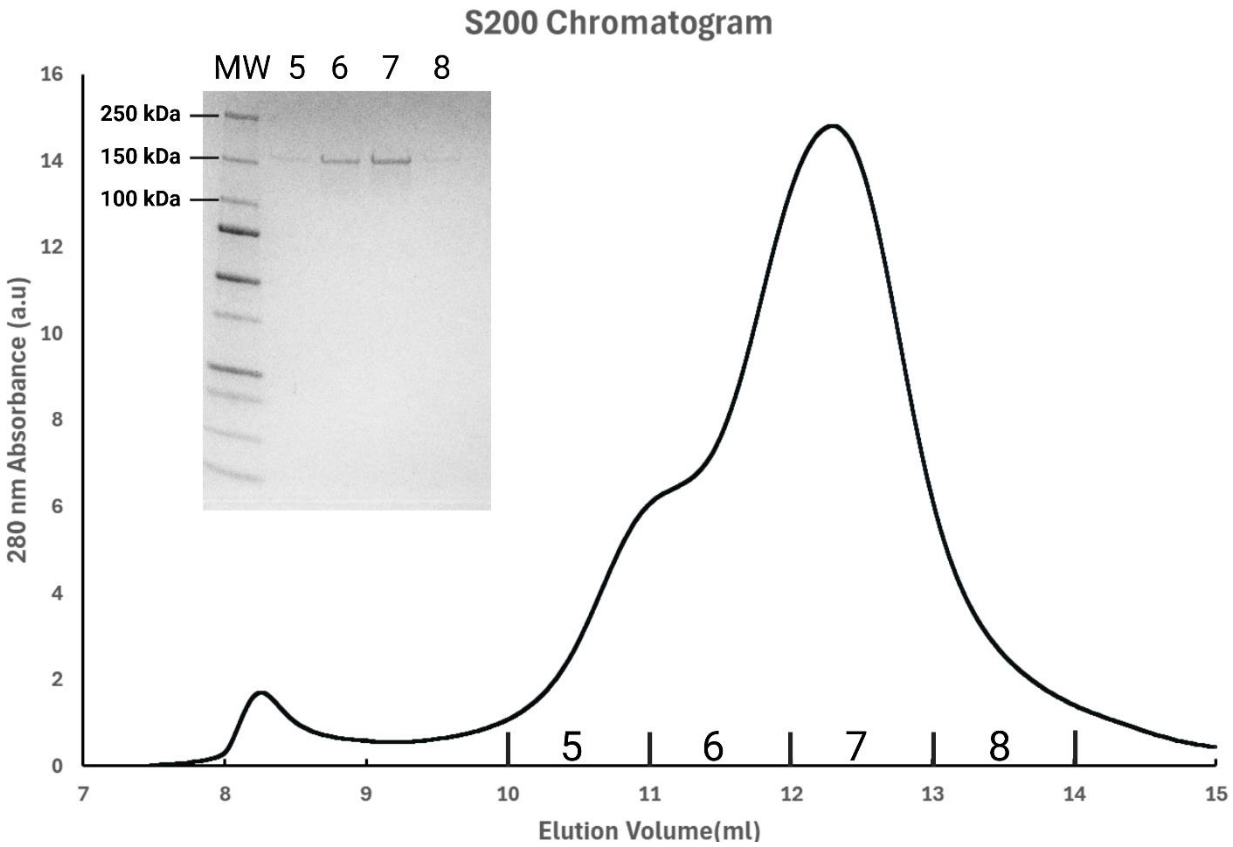

Figure S1. syNOS Purification. Representative chromatogram from a Superdex 200 increase 10/30 size exclusion chromatography column with an arrow denoting the syNOS dimer peak. The inset corresponds to a Coomassie blue stained 4-12% SDS-PAGE gel of fractions 5,6,7 and 8.

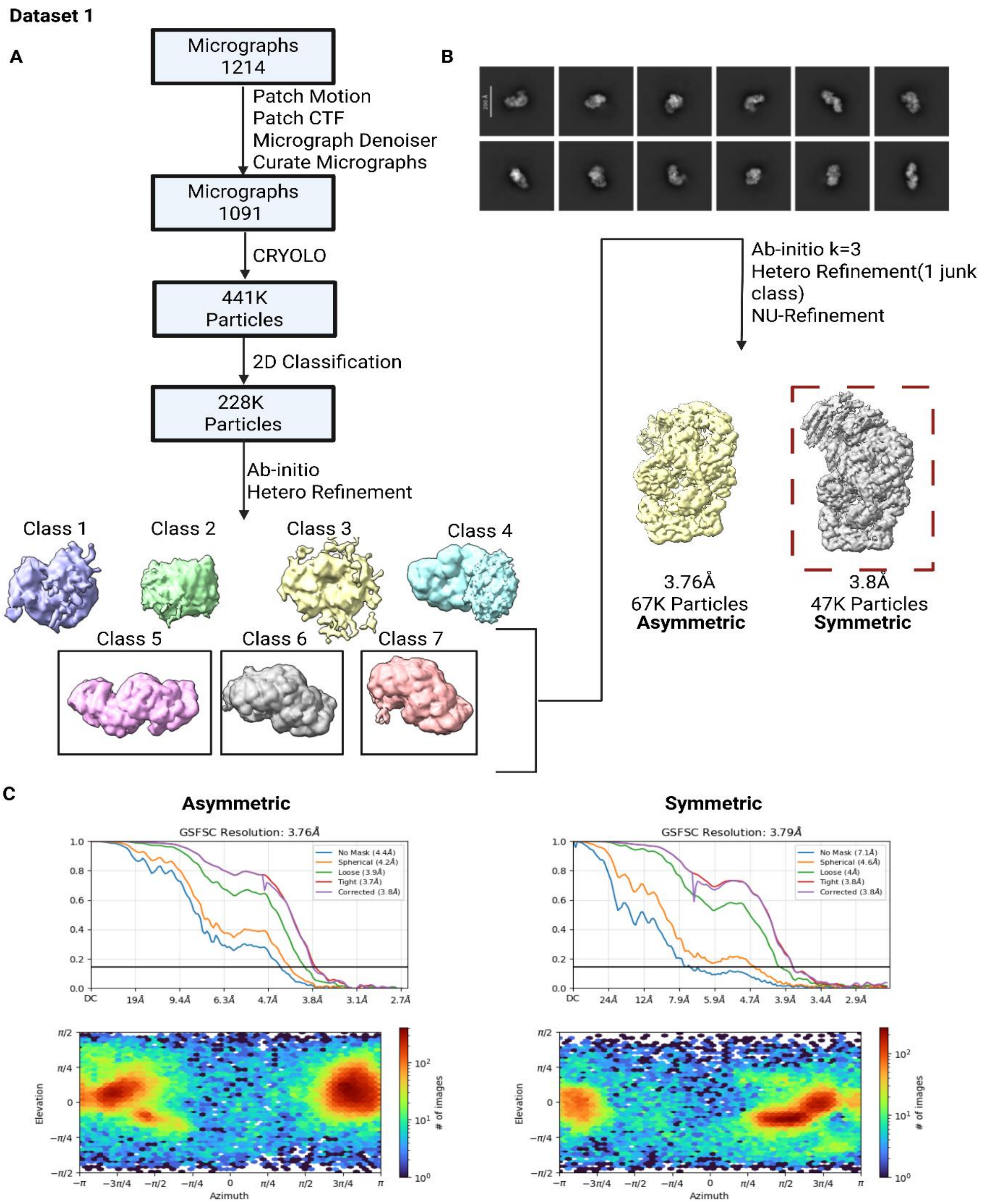

Figure S2. Dataset 1 processing scheme. (A) CryoEM processing workflow for dataset 1: syNOS ligand free. NOS-Sym class used for 3DVA and 3DFlex boxed (dashed-red). (B) Representative 2D Classes. CTF, contrast transfer function; NU, non-uniform refinement. (C) Overall Particle and

Gold-standard Fourier Shell Correlation (GSFSC) curves for NOS-Asym and NOS-Sym. Created in BioRender. Crane, B. (2026) https://BioRender.com/z0l5nni.

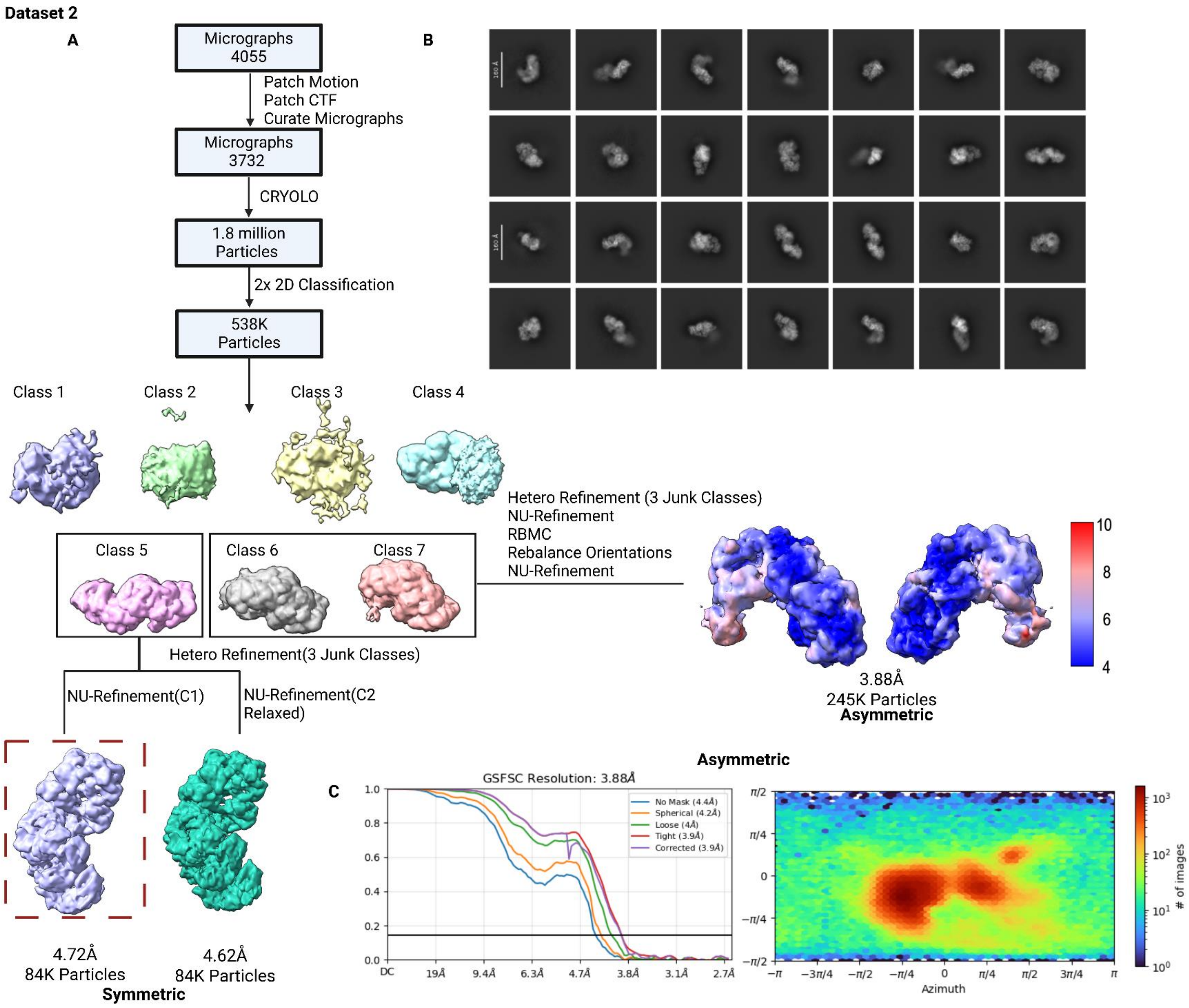

Figure S3. Dataset 2 processing scheme. (A) CryoEM processing workflow for dataset 2: syNOS ligand free. NOS-Sym class used for 3DVA and 3DFlex boxed (dashed-red). (B) Representative 2D

Classes. (C) GSFSC curves and positional distribution of particles. Created in BioRender. Crane, B. (2026) https://BioRender.com/z0l5nni.

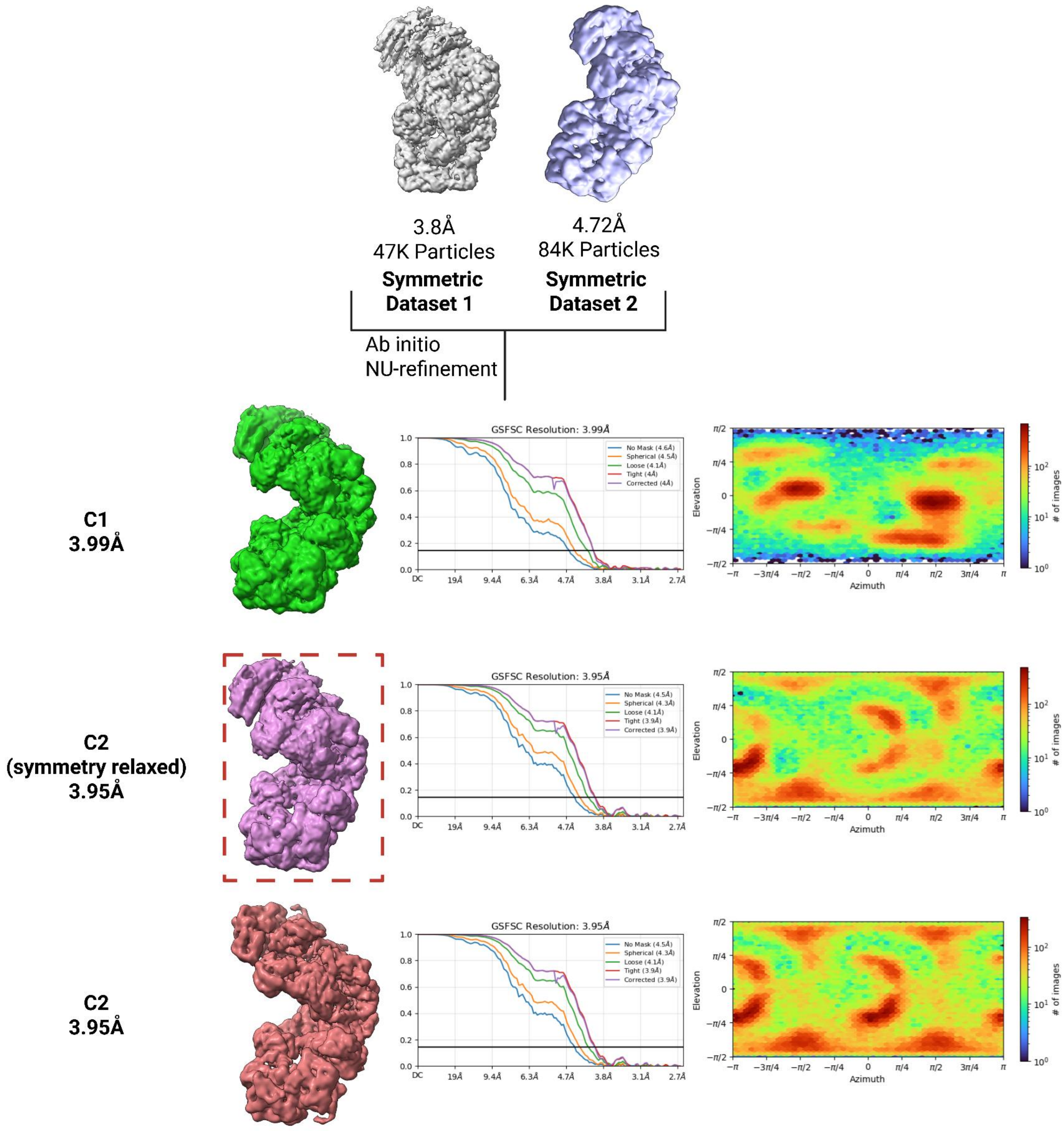

Figure S4. Merged data for NOS-Sym state, boxed class used for 3DVA and 3DFlex. Created in

BioRender. Crane, B. (2026) https://BioRender.com/z0l5nni.

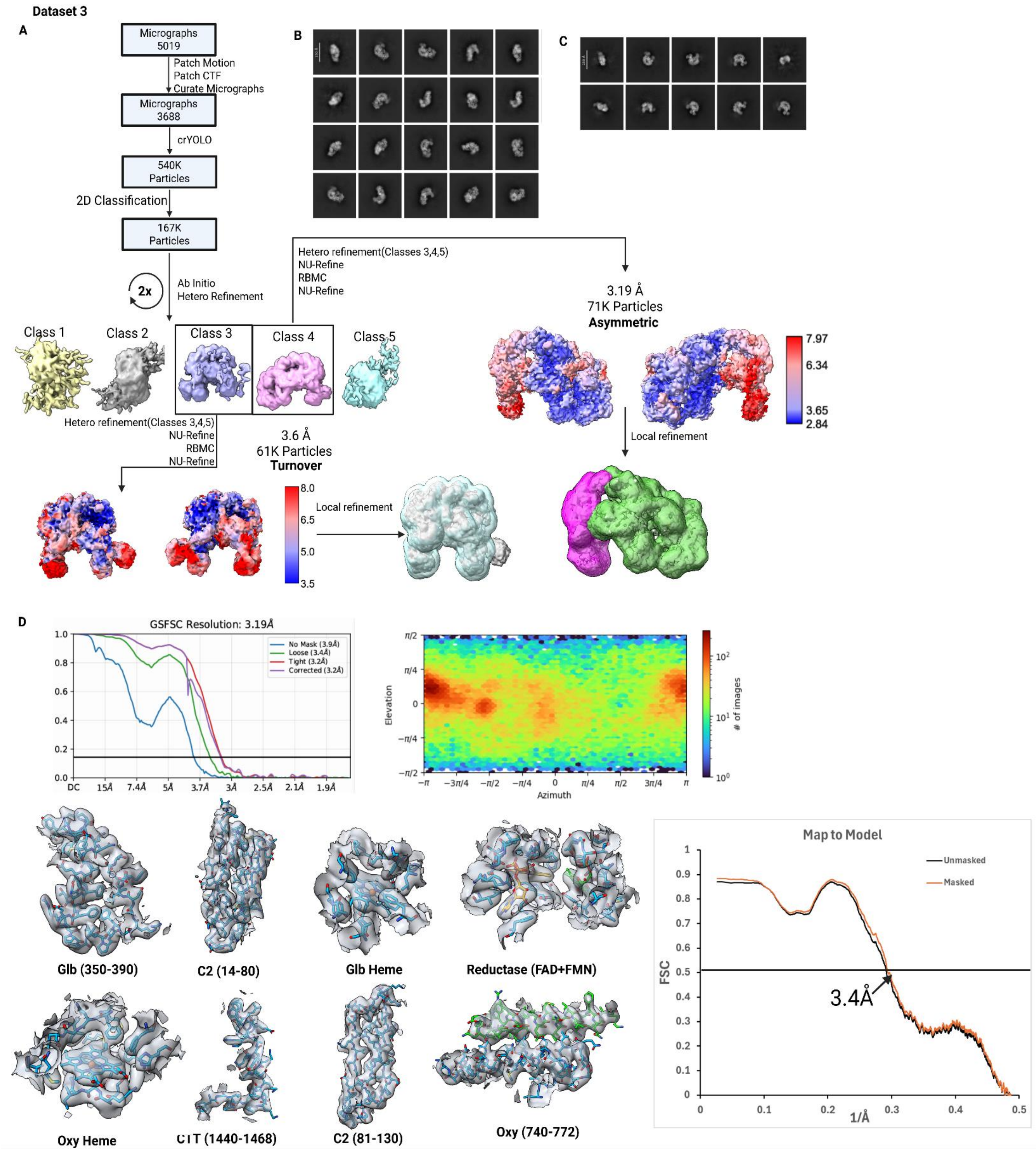

Figure S5. Dataset 3 processing scheme and validation. (A) CryoEM processing workflow for dataset 3: syNOS in the presence of Ca^{2+}/L-arginine (B) Representative 2D Classes for NOS-Asym

state (C) Representative 2D classes for NOS turnover state (NOS-TO_L) (D) Asymmetric states GSFSC curves, orientation validation, model density fit and map to model Fourier Shell Correlation (FSC). Created in BioRender. Crane, B. (2026) https://BioRender.com/n07x40q.

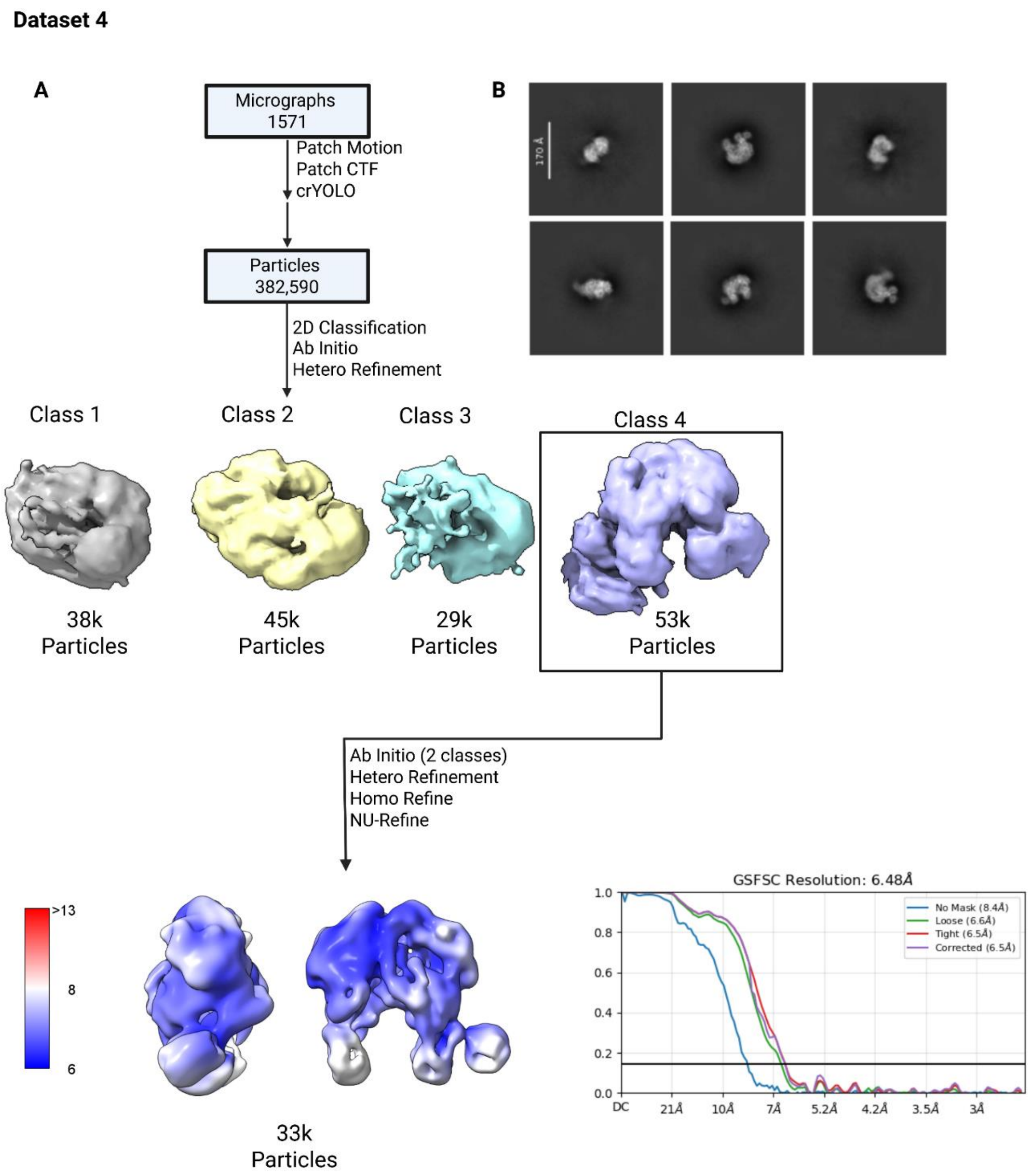

Figure S6. Dataset 4 processing scheme and validation. (A) CryoEM processing workflow for

dataset 4, syNOS with NADPH/Ca^{2+} including GSFSC curves. (B) Representative 2D classes for NOS-TO_T. Created in BioRender. Crane, B. (2025) https://BioRender.com/jwghotx.

Dataset 5

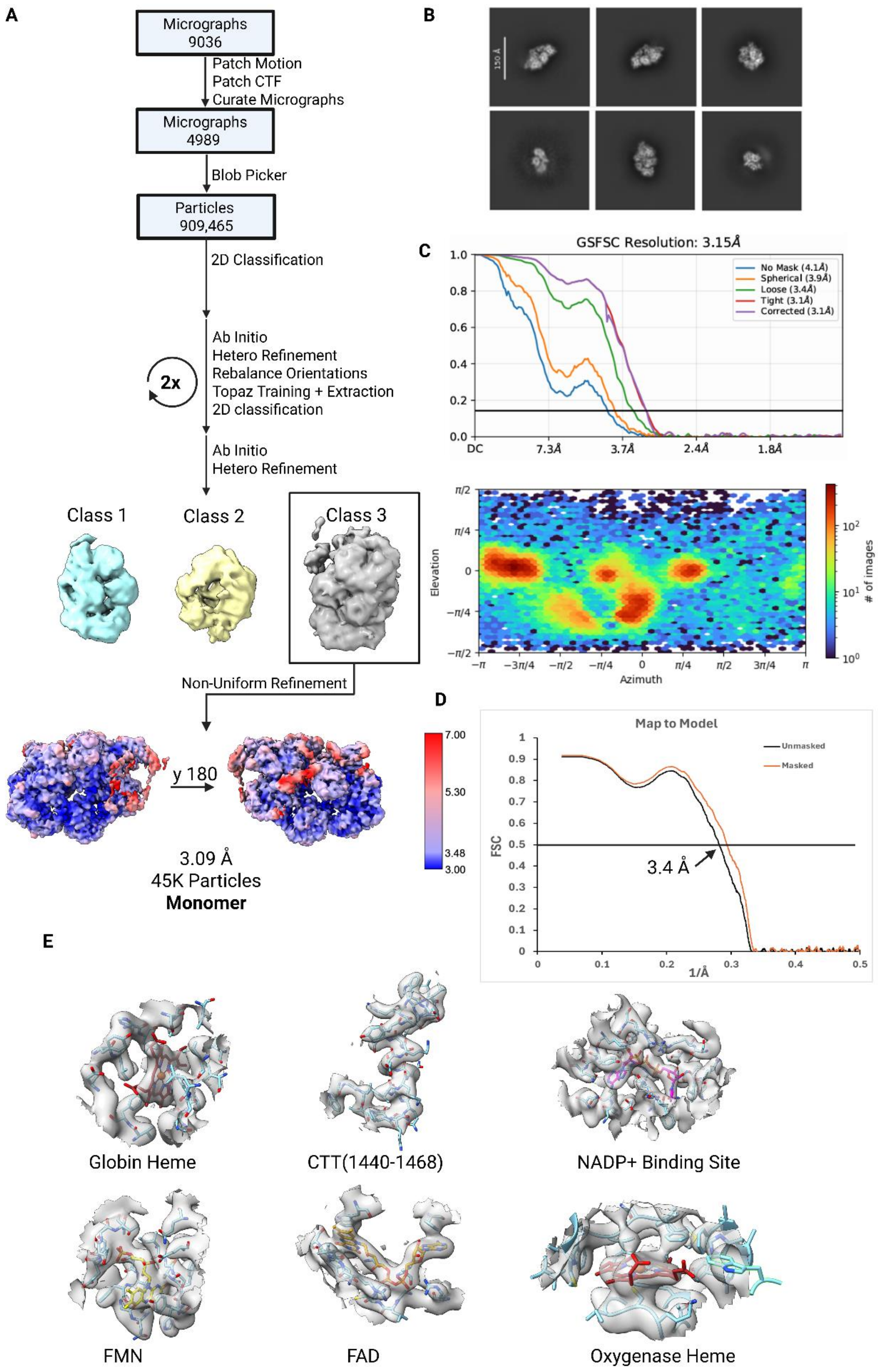

A
Micrographs 9036
Patch Motion
Patch CTF
Curate Micrographs
Micrographs 4989
Blob Picker
Particles 909,465
2D Classification
2x
Ab Initio
Hetero Refinement
Rebalance Orientations
Topaz Training + Extraction
2D classification
Ab Initio
Hetero Refinement
Class 1
Class 2
Class 3
Non-Uniform Refinement
y 180
3.09 Å
45K Particles
Monomer
7.00
5.30
3.48
3.00
B
150 Å
C
GSFSC Resolution: 3.15Å
No Mask (4.1Å)
Spherical (3.9Å)
Loose (3.4Å)
Tight (3.1Å)
Corrected (3.1Å)
DC
7.3Å
3.7Å
2.4Å
1.8Å
Elevation
Azimuth
# of images
D
Map to Model
Unmasked
Masked
FSC
3.4 Å
1/Å
E
Globin Heme
CTT(1440-1468)
NADP+ Binding Site
FMN
FAD
Oxygenase Heme

Figure S7. **Dataset 5 processing scheme and validation.** (A) CryoEM processing workflow for dataset 5: syNOS with L-arginine, NADPH/Ca^{2+}/NADPH (B) Representative 2D classes for NOS-Mon. (C) GSFSC curves, orientation validation and (D) Map to Model FSC. (E) Density fit of model in map. Created in BioRender. Crane, B. (2026) https://BioRender.com/xc3b4w7.

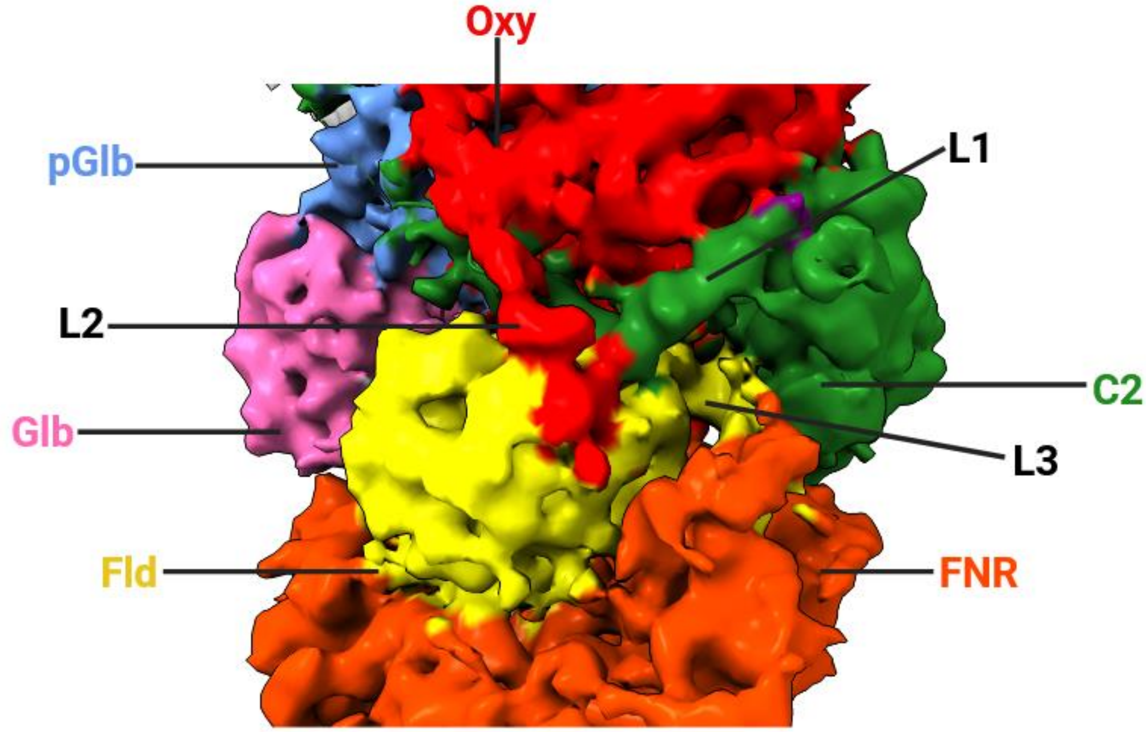

Figure S8. SyNOS linker intertwining. Intermediate resolution homodimer cryoEM density with all three linkers colored the same as their preceding domains. Note how L1 threads beneath L2 and on top of L3. Movement of the C2 domain (green) will release the Fld module for NOS_{Oxy} reduction. Created in BioRender. Crane, B. (2026) https://BioRender.com/yvw83aw

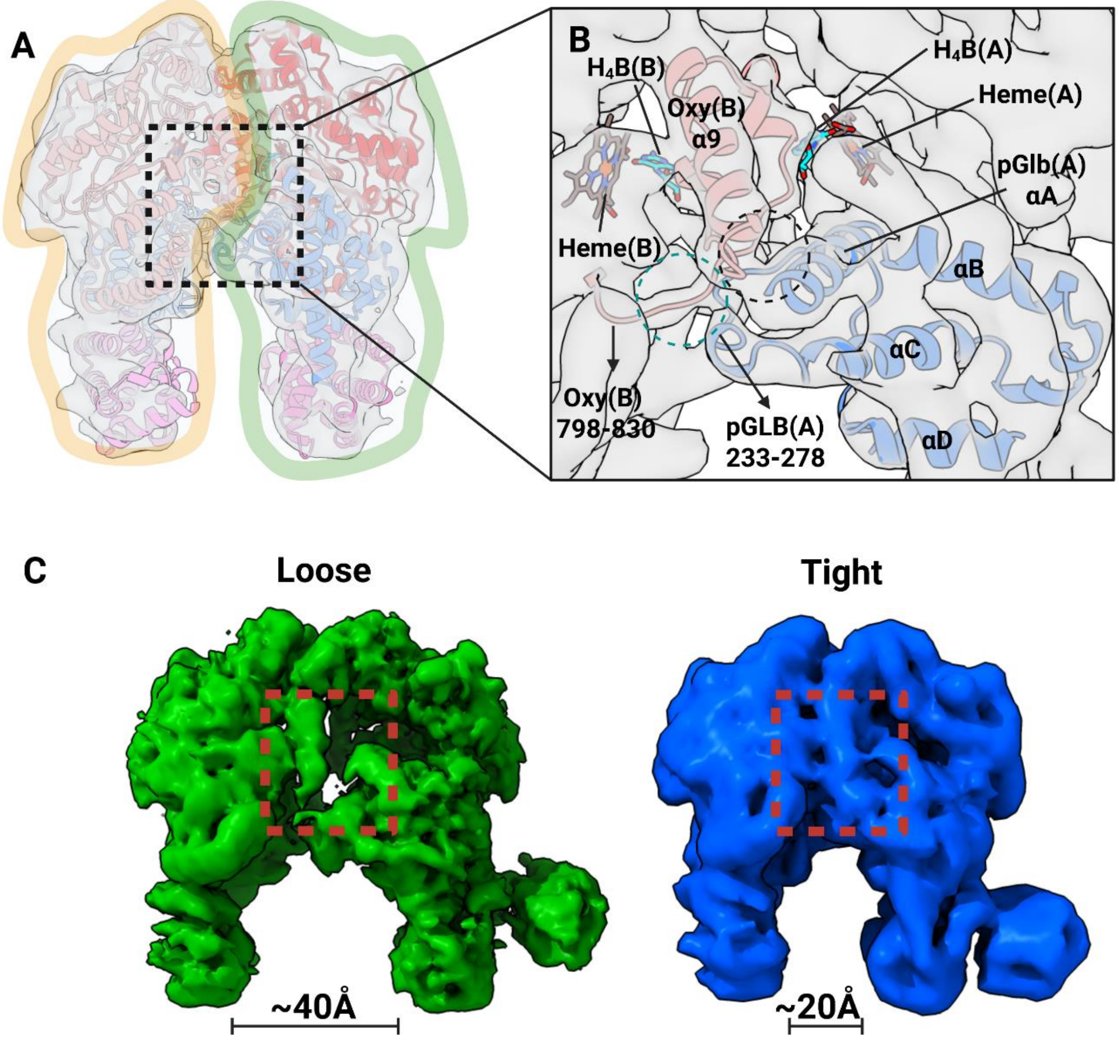

Figure S9. SyNOS tight and loose NOS_{Oxy} states. (A) syNOS cryoEM density (dataset 4) with the NOS_{Oxy} dimer interface outlined (orange and green) to show each subunit. (B) Close up of the dimeric interface showing the close association of NOS_{Oxy} α9 to an intersubunit pGlb C/D loop. Although at lower resolution, strong electron density for the core the NOS-TO_T interface includes the helical lariats and BH_4. (C) Loose (green) and tight (blue) NOS_{Oxy} dimers differ by a large 20 Å breathing motion normal to the dimer interface that also affects the positioning of the C2 domain (lower right peripheral electron density). Created in BioRender. Crane, B. (2026) https://BioRender.com/murzddq.

A

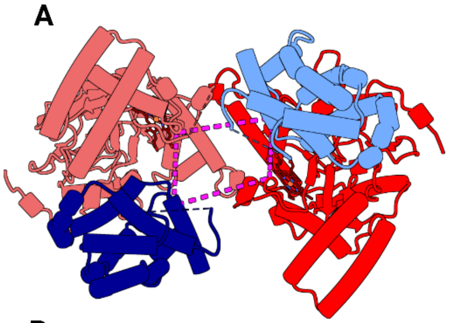

Inactive Dimer Interface

Residue 1	Residue 2	Intra Cα- Cα	Inter Cα- Cα	XlinkX Score
191(pGlb)	538(Oxy)	**17**	**24**	78

B

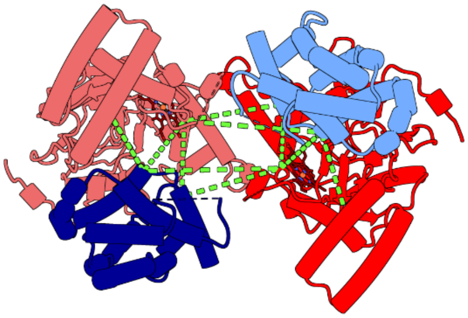

Turnover Dimer Interface

Residue 1	Residue 2	Intra Cα- Cα	Inter Cα- Cα	XlinkX Score
196(pGlb)	538(Oxy)	**16**	**31**	97
191(pGlb)	538(Oxy)	**17**	**24**	78
196(pGlb)	481(Oxy)	**24**	**44**	48

C

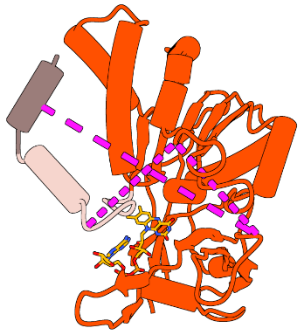

Inactive CTT

Residue 1	Residue 2	Intra Cα- Cα	Inter Cα- Cα	XlinkX Score
1366	1447(CTT)	**20**	117	118
1053	1463(CTT)	**40**	121	75
1366	1053(CTT)	**21**	138	52

Figure S10. DSBU crosslinks plotted onto the dimer interface of syNOS Oxy + pGlb and the syNOS FNR + CTT. (A) syNOS inactive dimer interface, subunit 1 Oxy (red) + pGlb (blue) and subunit 2 Oxy (bright red) + pGlb (navy), with magenta crosslinks corresponding to table (left). (B) syNOS inactive dimer interface, protomer 1 Oxy (red) + pGlb (blue) and subunit 2 Oxy (bright red) + pGlb (navy), with green crosslinks corresponding to table (left). (C) syNOS inactive CTT with magenta crosslinks corresponding to table (left). Crosslinks within range based on the structures are underlined and bolded. Created in BioRender. Crane, B. (2026) https://BioRender.com/2qt3ecc.

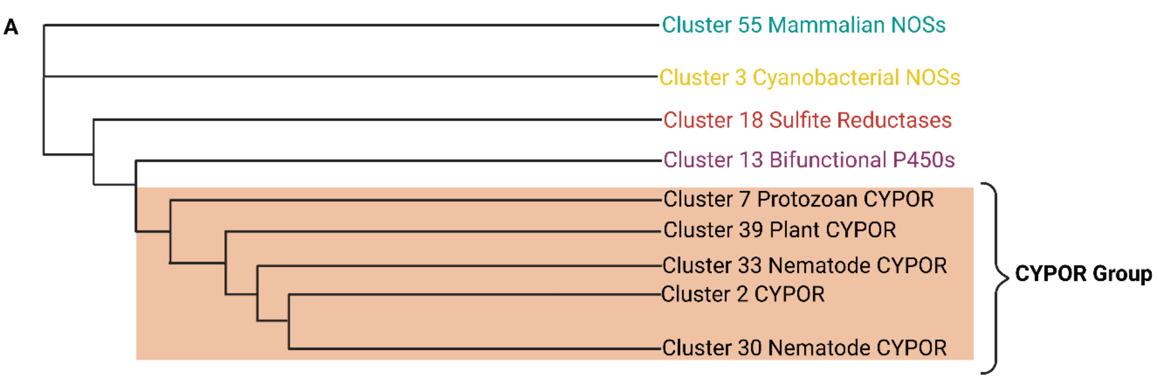

B

Cluster ID	Average Sequance Length	Standard Deviation of Sequence Length	Sequence Count
2	678	63	2521
39	702	49	1588
55	1117	103	278
3	1479	118	94
13	976	174	63
58	700	17	49
30	1126	283	47
7	638	35	45
18	601	74	44
33	652	36	44

Figure S11. Phylogenetic sequence clustering in the context of syNOS$_{Red}$(residues 853-1468). Representative clusters were subjected to PhyML with clusters above 40 sequences represented in the tree. (B) Cluster composition by sequence length and count. Created in BioRender. Crane, B. (2026) https://BioRender.com/ullben1.

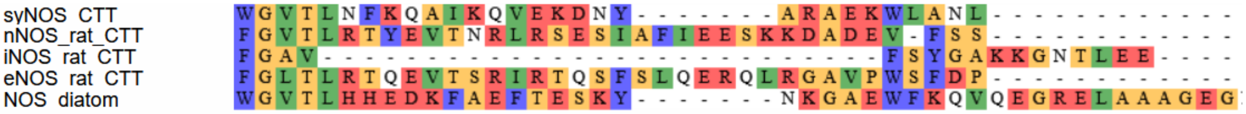

Figure S12. C-terminal tail (CTT) sequence alignment of syNOS, mNOSs and diatom NOS.

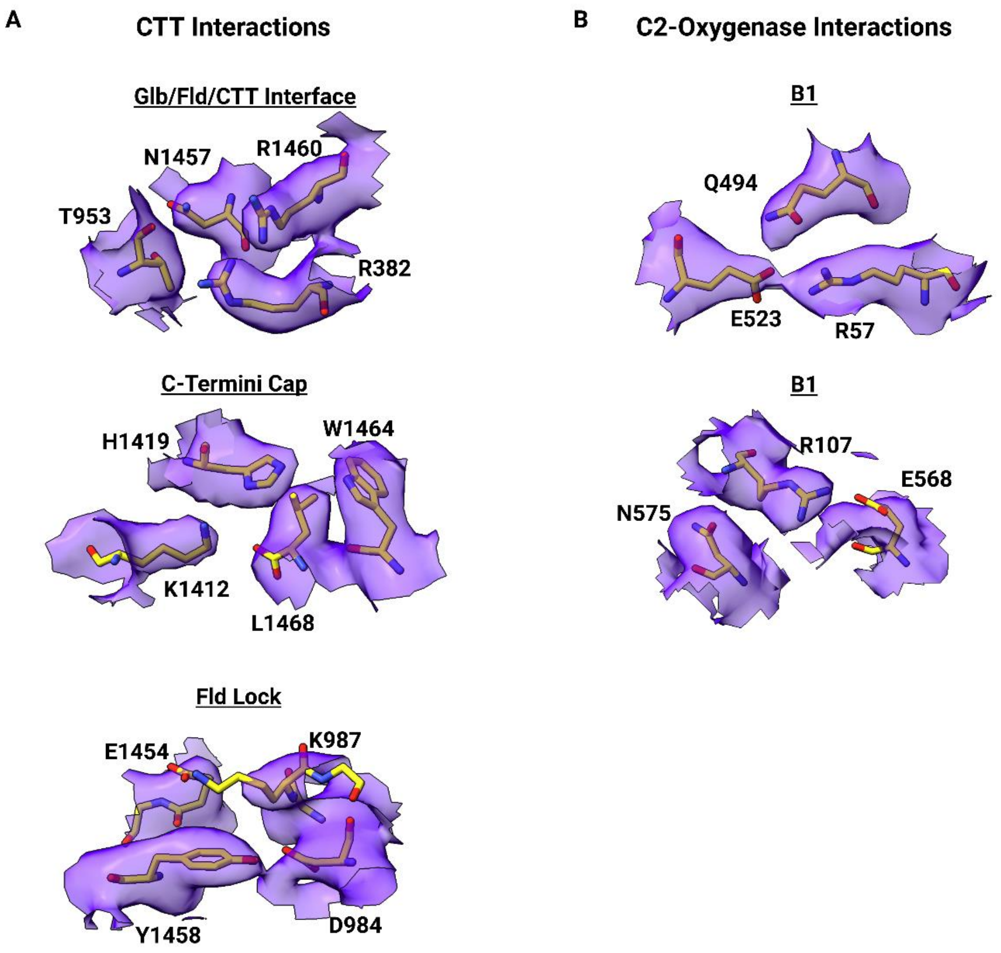

Figure S13. **Cryo-EM density validation of key interactions.** (A) Density fit of syNOS-Asym (9Q15), key interactions shown in Fig 3 C-E (B) Density fit of syNOS-Asym (9Q15), key interactions shown in Fig 4. B1-B2. Created in BioRender. Crane, B. (2026) https://BioRender.com/147lf5l.

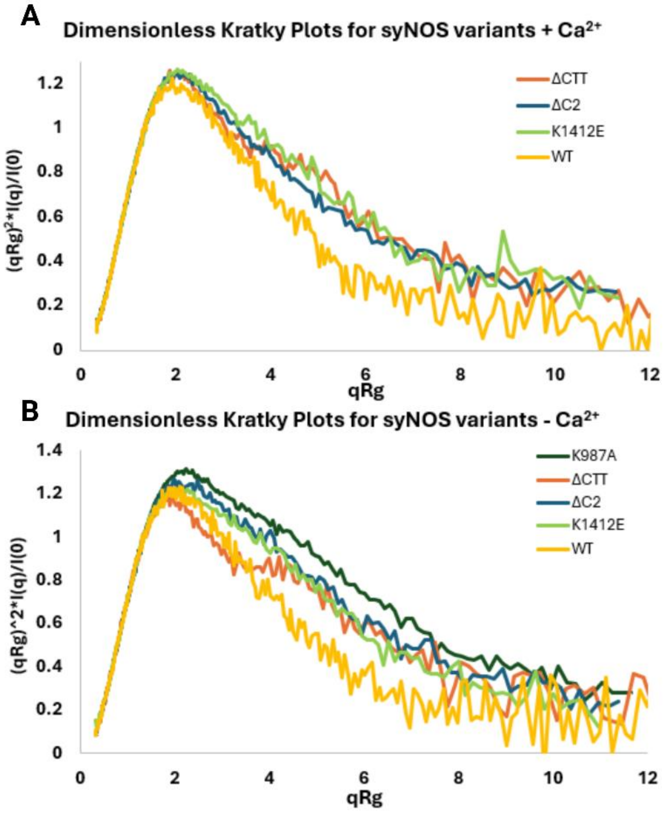

C

Sample	Rg [Guinier] (Å)	MW(Vc)	MW(Vp)
Predicted WT Dimer	55.59	-	-
Predicted WT Monomer	38.81	-	-
WT Ca^{2+}	52.83 ± 0.44	277.8	316.8
ΔC2 Ca^{2+}	56.89 ± 0.29	317.9	366.9
ΔCTT Ca^{2+}	62.71 ± 0.37	237.9	288.6
K1412E Ca^{2+}	57.16 ± 0.3	222	284
WT EGTA	52.83 ± 0.44	234.7	280.2
ΔC2 EGTA	57.34 ± 0.3	222.7	272.2
ΔCTT EGTA	61.18 ± 0.55	313.3	360
K1412E EGTA	55.28 ± 0.24	221.4	258.2
K987A EGTA	58.79 ± 0.37	213.6	266.7

Figure S14. SEC-SAXS of syNOS and variants. (A) Dimensionless Kratky plots, binned by 4 qRg values, for samples in the presence and absence of Ca^{2+}. (B) Table of predicted and experientially characterized radius of gyrations (Rgs) and molecular weights calculated either by volumes of correlation (V_C) or Porod volumes (V_P). (Samples were measured once) Created in BioRender. Crane, B. (2026) https://BioRender.com/0p6iuvd.

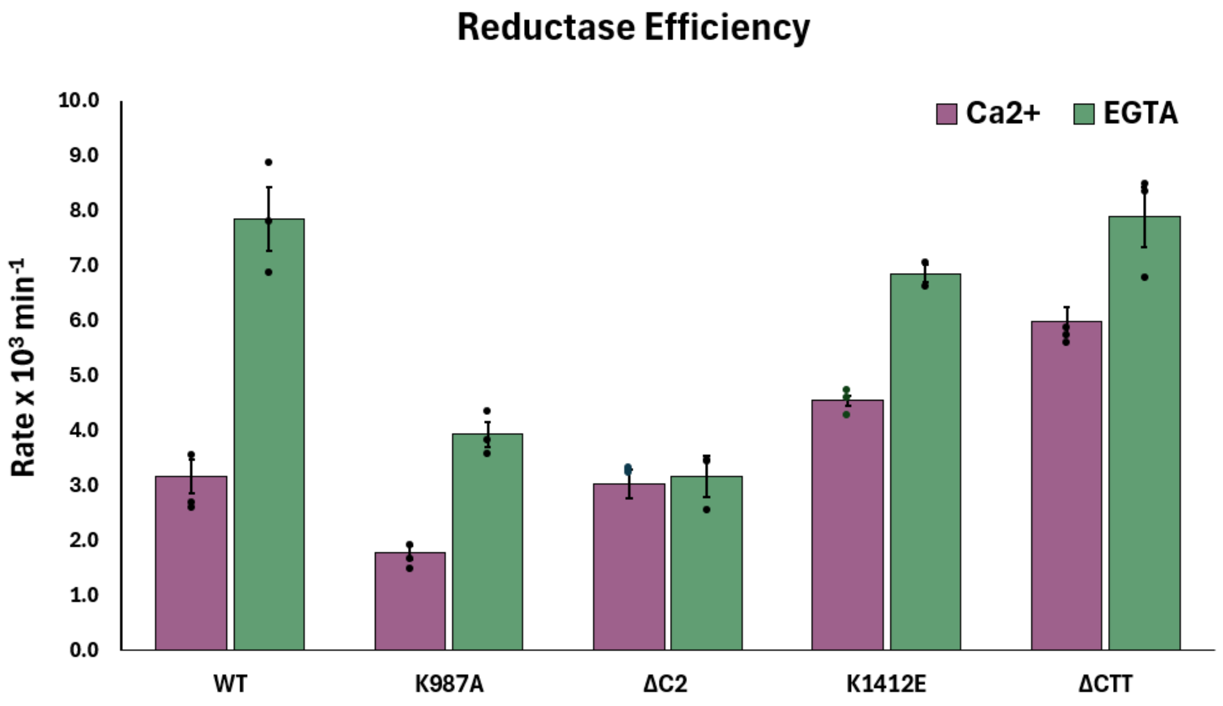

Figure S15. Steady state cytochrome c reductase activities of WT syNOS and variants expressed as turnover numbers (Cc reduced (NOS subunit)$^{-1}$ min^{-1}) with standard errors shown as error bars (see Methods). Statistical comparisons between groups were performed with a 2-way ANOVA with post hoc analysis using Tukey's multiple comparisons test. Significant differences are indicated as follows: ns (not significant), * ($p \leq 0.05$), ** ($p \leq 0.01$), *** ($p \leq 0.001$), **** ($p \leq 0.0001$)..Ca:WT vs. Ca:K987A (ns); Ca:WT vs. Ca:ΔC2 (ns); Ca:WT vs. Ca:K1412E (ns); Ca:WT vs. Ca:ΔCTT (ns); Ca:WT vs. EGTA:WT (****) ; Ca:WT vs. EGTA:K987A (ns); Ca:WT vs. EGTA:ΔC2 (ns); Ca:WT vs. EGTA:K1412E (****); Ca:WT vs. EGTA:ΔCTT (****) ;Ca:K987A vs. Ca:ΔC2 (ns); Ca:K987A vs. Ca:K1412E (***); Ca:K987A vs. Ca:ΔCTT (****); Ca:K987A vs. EGTA:WT (**); Ca:K987A vs. EGTA:K987A (**); Ca:K987A vs. EGTA:ΔC2 (ns); Ca:K987A vs. EGTA:K1412E (****); Ca:K987A vs. EGTA:ΔCTT (****); Ca:ΔC2 vs. Ca:K1412E (ns); Ca:ΔC2 vs. Ca:ΔCTT (**); Ca:ΔC2 vs. EGTA:WT (****); Ca:ΔC2 vs. EGTA:K987A (ns); Ca:ΔC2 vs. EGTA:ΔC2 (ns); Ca:ΔC2 vs. EGTA:K1412E (****); Ca:ΔC2 vs. EGTA:ΔCTT (****);Ca:K1412E vs. Ca:ΔCTT (ns); Ca:K1412E vs. EGTA:WT (****); Ca:K1412E vs. EGTA:K987A (ns); Ca:K1412E vs. EGTA:ΔC2 (ns); Ca:K1412E vs. EGTA:K1412E (**); Ca:K1412E vs. EGTA:ΔCTT (****); Ca:ΔCTT vs. EGTA:WT (**); Ca:ΔCTT vs. EGTA:K987A (*); Ca:ΔCTT vs. EGTA:ΔC2 (***); Ca:ΔCTT vs. EGTA:K1412E (ns);

Ca:ΔCTT vs. EGTA:ΔCTT (**); EGTA:WT vs. EGTA:K987A (****); EGTA:WT vs. EGTA:ΔC2 (****); EGTA:WT vs. EGTA:K1412E (ns); EGTA:WT vs. EGTA:ΔCTT (ns); EGTA:K987A vs. EGTA:ΔC2 (ns); EGTA:K987A vs. EGTA:K1412E (***); EGTA:K987A vs. EGTA:ΔCTT (***); EGTA:ΔC2 vs. EGTA:K1412E (****); EGTA:ΔC2 vs. EGTA:ΔCTT (****); EGTA:K1412E vs. EGTA:ΔCTT (ns). (n=3, except for K1412E +EGTA; n = 2).

A

Reductase	λ_{obs}	A_1 (%)	k_{obs1} (s^{-1})	A_2 (%)	k_{obs2} (s^{-1})	A_3 (%)	K_{obs3} (s^{-1})	
$syNOS_{Red}(-Ca^{2+})$	450	89	178 (± 0.78)	11	4.12 (± 0.03)	-	-	This study
$syNOS_{FL}(-Ca^{2+})$	450	63	2.38 (± 0.03)	37	0.004 (± 0.001)	-	-	This study
$nNOS_{Red}$(-CAM)	458	28	57 (± 1)	52	5.2 (± 0.1)	20	570 (± 41)	Craig et al.[17]
$nNOS_{Red}$(+CAM)	458	44	608 (± 66)	56	86 (± 2)	-	-	Craig et al.[17]
CYPOR	450	90	42 (± 4)	10	3.8 (± 0.5)	-	-	Hamdane et al.[68]

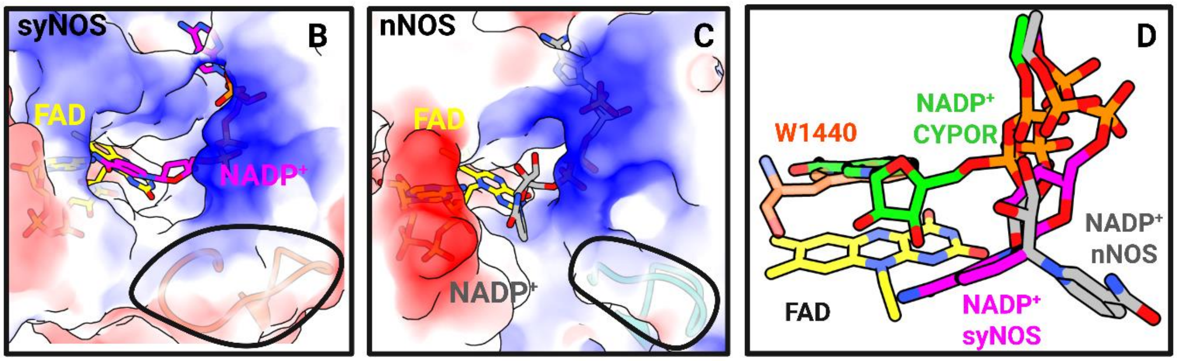

Figure S16. Flavin reduction by NADPH. (A) Table of flavin reduction rate constants for syNOS, nNOS and CYPOR(n = 4). (B) Surface representation and model of $NADP^+$ (magenta) bound in the pocket of syNOS-Mon with loop 1050-1062 (orange-red and circled in black) in comparison to (C) nNOS bound to $NADP^+$ (grey) with corresponding loop 1002-1012 (aqua) circled Iin black. (D) Overlay of $NADP^+$ bound conformations of syNOS, nNOS and CYPOR W677X (1-676) (PDB: 1JA0) (green) over a consensus position of FNR-bound FAD (yellow). syNOS CTT residue 1440 blocks access of the nicotinamide ring to the isoalloxazine ring. Created in BioRender. Crane, B. (2025) https://BioRender.com/g79rbtd.

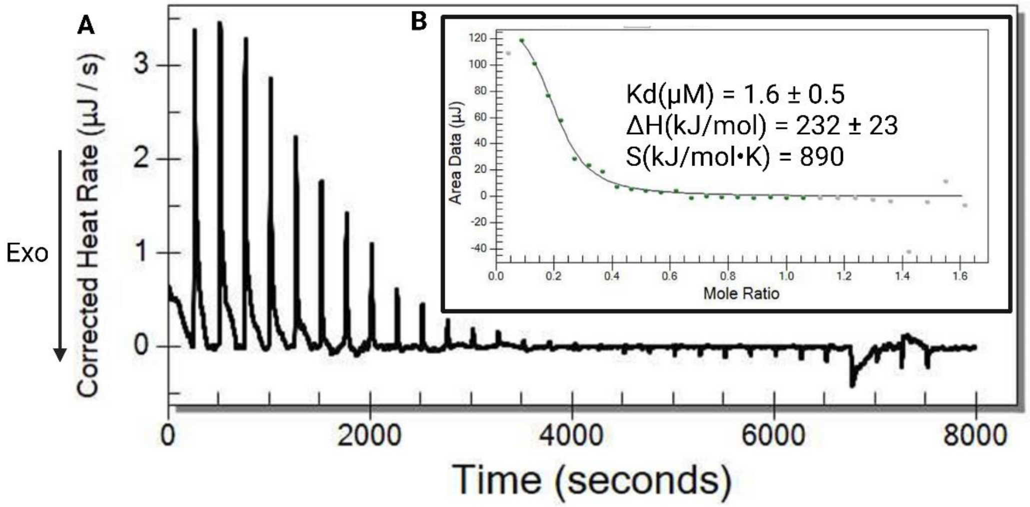

Figure S17. Isothermal calorimetric titration of Ca^{2+} with $syNOS_{C2}$ (residues 1-138). (A) Endothermic, baseline corrected isotherm of Ca^{2+} titrated into $syNOS_{C2}$. (B) Binding model fit with the accompanying thermodynamic parameters. (Samples were measured once) Created in BioRender. Crane, B. (2026) https://BioRender.com/hrdb5r4.

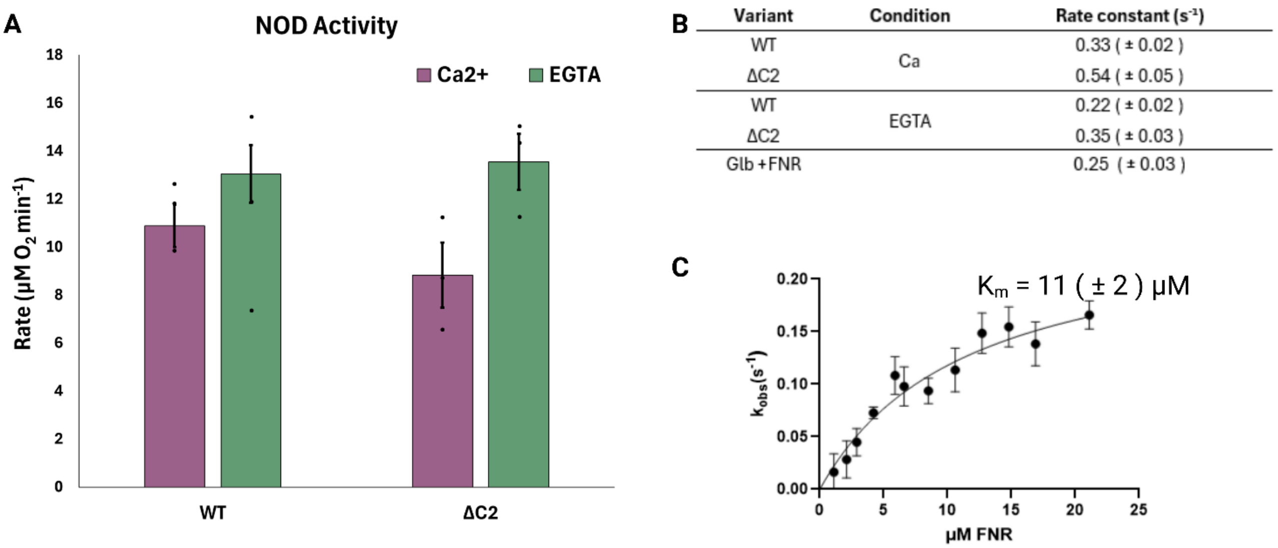

Figure S18. SyNOS globin-based NOD activity and heme reduction rates. (A) NOD activity was measured by O2 consumption in the presence of the NO-donor NOC-7 and NADPH for WT and $syNOS_{\Delta C2}$ (n=3, except for $syNOS_{\Delta C2}$ + Ca^{2+} ; n = 2). (B) Heme reduction rate constants of $syNOS_{Glb}$ in the presence or absence of Ca^{2+} for WT or $syNOS_{\Delta C2}$ and the truncated $syNOS_{Glb}$/$syNOS_{FNR}$ couple in the absence of Ca^{2+}, with standard error represented. Statistical comparisons between groups were performed with a 2-way ANOVA with post hoc analysis using Tukey's multiple comparisons test, all comparisons were non-significant. (C) Michelis-Menten plot of truncated $syNOS_{Glb}$ reduced by varied concentrations of truncated $syNOS_{FNR}$ until saturation, with standard deviation represented. (Each data point reflects at least n = 3.) Created in BioRender. Crane, B. (2026) https://BioRender.com/8dxm1yw.

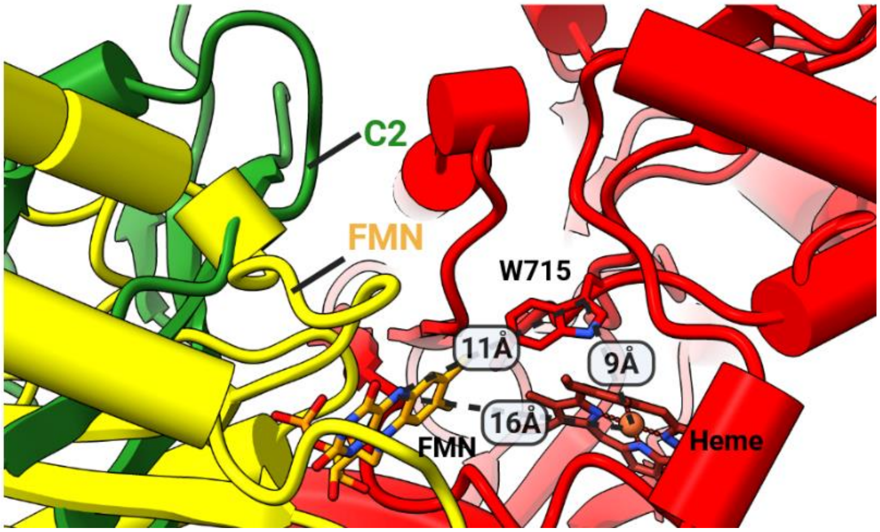

Figure S19. Output state predicted by AlphaFold3 with FMN-to-Trp715-to-Heme distances shown. Overlay of the C2 in the locked state (green) with the output Fld conformation (yellow) illustrates that the Fld is sterically blocked by the C2 domain in the NOS-Asym state. The AF3 prediction was made with a minimal unit of two truncated syNOS chains: 1) residues 473-863 (Oxy) and 2) residues 473-1003 (Oxy + Fld). Including additional domains did not predict interaction between $syNOS_{Fld}$ and $syNOS_{Oxy}$. Created in BioRender. Crane, B. (2026) https://BioRender.com/tf16vbu.
